# Opioid use disorder medication treatment in residential settings increased 2017-2021, but sustaining it following stays remains an issue

**DOI:** 10.1093/haschl/qxag175

**Published:** 2026-08-10

**Authors:** Stephan R Lindner, Brynna Manibusan, Kyle Hart, Dennis McCarty, Daniel Hartung, K John McConnell

**Affiliations:** Center for Health Systems Effectiveness (CHSE), Oregon Health & Science University (OHSU), 3030 S Moody Ave, Portland, OR 97201, United States; OHSU-PSU School of Public Health, 1810 SW 5th Avenue, Portland, OR 97201, United States; Center for Health Systems Effectiveness (CHSE), Oregon Health & Science University (OHSU), 3030 S Moody Ave, Portland, OR 97201, United States; Center for Health Systems Effectiveness (CHSE), Oregon Health & Science University (OHSU), 3030 S Moody Ave, Portland, OR 97201, United States; Division of General and Internal Medicine, Oregon Health & Science University (OHSU), 3181 SW Sam Jackson Park Road, Portland, OR 97239, United States; College of Pharmacy, Oregon State University, 1601 SW Jefferson Ave, Corvallis, OR 97331, United States; Center for Health Systems Effectiveness (CHSE), Oregon Health & Science University (OHSU), 3030 S Moody Ave, Portland, OR 97201, United States; OHSU-PSU School of Public Health, 1810 SW 5th Avenue, Portland, OR 97201, United States

**Keywords:** residential treatment, OUD medication treatment, SUD waiver

## Abstract

**Introduction:**

Residential treatment is part of the care continuum for opioid use disorder (OUD). However, use of OUD medications (MOUD) has been historically low in this setting. The goal of this study was to assess changes in MOUD during residential treatment stays and the 12-month period surrounding them.

**Methods:**

We used national Medicaid claims data for the year 2017-2021 and descriptive as well as regression analyses.

**Results:**

Between 2017 and 2021, the rate of MOUD treatment in residential settings increased from 30.4 to 58.5 percentage points. By contrast, the rate of MOUD treatment 6 months before residential treatment stays and 5 months following residential treatment stays was low in 2017 and increased only slightly. Changes were similar in states that implemented a substance use disorder waiver compared to non-waiver states. Most states exhibited similar changes. New York was the only state where MOUD rates before and after residential treatment stays increased markedly.

**Conclusions:**

Our study suggests that use of MOUD during residential treatment stays increased substantially 2017-2021, but that this increase was not sustained during the months following stays. Improving retention in MOUD treatment following residential treatment stays could be an important future policy goal.

Key Points

- The rate of medication treatment for opioid use disorder (MOUD) increased substantially during residential treatment stays among Medicaid enrollees 2017-2021.
- By contrast, rates of MOUD were low 5 months following stays and increased only slightly.
- Thus, improving retention in MOUD treatment following residential treatment stays could be an important future policy goal.

## Introduction

Residential treatment is part of the continuum of care for the treatment of opioid use disorders (OUDs).^1^ Medication for opioid use disorder (MOUD) (ie, buprenorphine, methadone, naltrexone) can minimize craving and withdrawal symptoms, stabilize patients, reduce the risk of opioid overdose, and enhance the effectiveness of residential and outpatient addiction treatment services.^2-4^ However, MOUD treatment in residential settings has historically been uncommon. In 2017, less than half of residential treatment facilities offered MOUD, and only 15% of patients with OUD received an MOUD as part of their treatment plan.^5^ The lack of medication access in residential settings has been a particular concern for Medicaid programs, which cover a disproportionate share of people with OUD.

Two important developments over the last decade may have contributed to an increased use of MOUD in residential settings. First, the use of MOUD has increased steadily among the Medicaid population, influenced by policy initiatives such as coverage changes for methadone or removal of prescribing barriers for buprenorphine.^6-8^ While these policies did not specifically target the residential treatment setting, they may nonetheless have contributed to increased acceptance and use of MOUD in this setting.

The second development is the continued adoption of Section 1115 substance use disorder (SUD) waivers. These waivers allow states to receive additional federal funding but require them to make various changes to their Medicaid program.^9,10^ One such requirement stipulates that residential treatment facilities have to offer MOUD either on-site or to facilitate access off-site. Studies on SUD waivers reported a higher rate of residential treatment stays in waiver states compared to non-waiver states prior to waiver implementation (3.9% in waiver states vs 0.9% in non-waiver states during the second quarter of 2016); moreover, they found that SUD waiver implementation was associated with an increase in residential treatment facilities accepting Medicaid patients and providing OUD medication treatment, and in Medicaid enrollees using this type of service.^11-13^ However, it is not known whether the use of OUD medications in residential settings has increased over time as a result of waivers.

To address these gaps in knowledge, we sought to (1) assess changes in MOUD treatment in residential settings; (2) examine whether implementation of SUD waivers was associated with increased use of MOUD in residential settings over time; and (3) investigate variation in changes across states. We used Medicaid records from 24 states to identify residential treatment stays between 2017 and 2021 and then examined changes in MOUD surrounding these residential treatment stays over time and across states. Specifically, we examined OUD medication treatment rates before, during, and following residential treatment stays, which allowed us to investigate whether Medicaid members with OUD were more likely to access MOUD before residential treatment stays over time, whether they increasingly connected to MOUD while in residential care, and if so, whether they successfully continued MOUD following their residential treatment stay.

## Study data and methods

### Data source

We used the 2016-2021 Transformed Medicaid Statistical Information System (T-MSIS) Analytic Files (TAF).^14^ TAF included comprehensive enrollment and claims information of individuals enrolled in state Medicaid fee-for-service and managed care plans.

### Study population

We included Medicaid members, ages 18-64 with OUD related residential treatment stays. We used International Statistical Classification of Diseases and Related Health Problems, Tenth Revision codes F11.XXX to identify OUD diagnoses, and a combination of revenue, current procedural terminology (CPT), and place of service codes to capture residential treatment stays in TAF records, as described in prior studies.^15-18^

We excluded individuals with missing enrollment records during the 12-month period surrounding their residential treatment stays, from 6 months preceding the month during which the residential treatment stay began (ie, the month of the residential treatment stay) to 5 months following the month of the residential treatment stay. We also excluded individuals who (1) were dually eligible for Medicaid and Medicare because we did not have Medicare records, (2) were enrolled in more than 1 state during a month, or (3) received limited Medicaid benefits. Finally, we excluded Medicaid members residing in 2 states for which we identified data quality issues and in 19 states for which we observed fewer than 50 residential treatment stays during any year of the study period (see online Appendix Section A-1 for details) (To access the appendix, click on the Details tab of the article online.)

### Measures

Primary outcomes included binary measures of any MOUD treatment for each month during the 12-month period surrounding residential treatment stays and the number of months with any MOUD treatment during that period (range: 0-12). We used National Drug Codes, CPT codes, and Healthcare Common Procedure Coding System codes to capture MOUD treatment.^8^ Secondary outcomes included types of MOUD (buprenorphine, methadone, or naltrexone). We included multiple treatment stays for an enrollee as separate stays; we excluded stays that started in the same month as the previous stay for the same enrollee. Covariates in our analysis included age groups (18-24, 25-34, 35-44, 45-54, 55-64), sex (female, male), eligibility groups (disabled adults, non-disabled adults, expansion adults, pregnant women, youth), presence of other SUDs (eg, alcohol disorder or other drug disorders), and presence of mental health conditions (eg, psychotic disorders, mood disorders). We also classified states based on their 1115 SUD waiver status, distinguishing between states that did and did not adopt waivers during the study period 2017-2021.

### Analysis

We first descriptively analyzed rates of MOUD treatment during the 12-month period surrounding the month of residential treatment stays, stratified by study years. We then estimated 3 regression models to assess changes in MOUD treatment between 2017 and 2021, focusing on MOUD treatment rates (1) during the month of residential treatment stays, (2) 6 months preceding the month of residential treatment stays, and (iii) 5 months following the month of residential treatment. The first model included an indicator for the year 2021 along with covariates to estimate the average covariate-adjusted change between 2017 and 2021. The second model expanded on the first by adding an indicator for states' SUD waiver status and interacting the term with the 2021-year indicator to assess whether changes over this period differed between waiver and non-waiver states. Similarly, the third model expanded on the first one by adding state fixed effects and interacting them with the 2021-year indicator to estimate changes within individual states. We clustered standard errors at the state level, except for the third model, for which we clustered at the county level because clustering at the state level was not feasible. Appendix Section A-2 includes technical details of our analysis (To access the appendix, click on the details tab of the article online.)

In supplemental analyses, we (1) estimated regression models for types of MOUD as outcomes; (2) excluded 4 states with approved SUD waivers in 2017 (Virginia, New Jersey) and 2021 (Maine, Oregon) in regressions examining changes in waiver vs non-waiver states; (3) excluded New York from the analysis assessing changes in waiver and non-waiver states (see the Results section for an explanation); and (4) estimated staggered difference-in-differences regressions that used more precise information on the timing of SUD waiver implementation.

The Institutional Review Board classified the analysis as not human subjects research. We used the TAF checklist to report the use of data, the definition of the analytic sample, and the exclusion of states.^19^ We used R version 4.3.3 for all analyses.

### Limitations

This study had several limitations. First, TAF data quality may vary across states. We excluded some states based on our review of TAF data quality; however, we could not rule out that some data quality issues remained in states included in our study. Second, we may not have captured all residential stays of Medicaid members in claims records. For example, some states may use specific codes that we were unaware of, or some Medicaid members may have accessed residential care using other funding sources. Third, it was beyond the scope of this study to examine factors other than the SUD waiver that may have led to changes in MOUD treatment in residential settings.

## Results

There were 189 558 individuals with 382 478 residential stays in the sample (Appendix Section A-3 [To access the appendix, click on the Details tab of the article online.]). The average length of stay was 16.3 days, and 13.7% of residential treatment stays exceeded a month. A majority of stays were by male individuals and those under 34 years. More than two-thirds of stays involved members eligible for the program due to Medicaid expansion. Close to half of all stays were by individuals with comorbid mental health conditions, and more than 70% of stays were by individuals with 1 or more additional SUD. Most residential stays were in waiver states.

**Figure 1.**
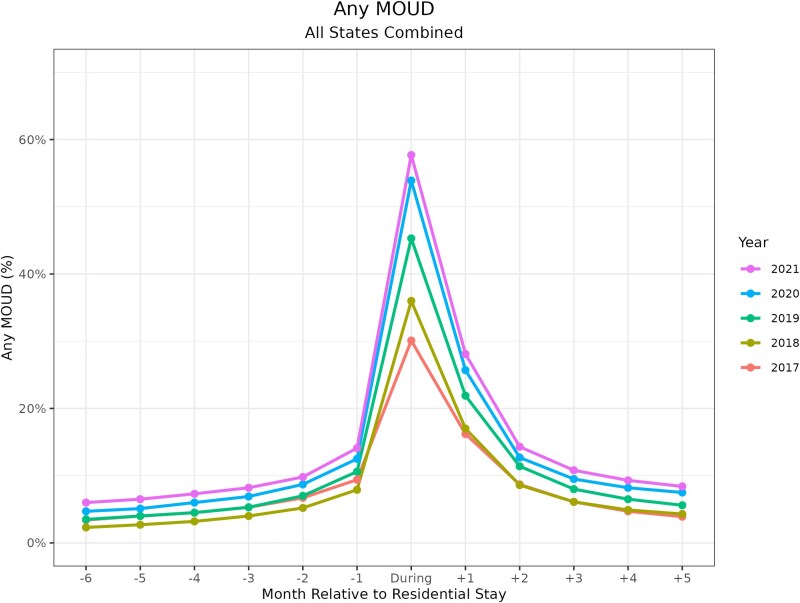
MOUD treatment relative to residential treatment stay. The denominator includes the number of Medicaid enrollees with an OUD who had a residential treatment stay and who were enrolled during the 12-month period surrounding the stay. Medicaid enrollees with several stays during the study period are included multiple times, 1 per stay. We excluded stays that occurred during the same month the previous stay started. The numerator for each month relative to the month of residential treatment stays was defined as the number of Medicaid enrollees included in the denominator with at least 1 MOUD claim during the respective month. Source: Transformed Medicaid Statistical Information System Analytic Files 2016-2021. Abbreviations: MOUD, medication for opioid use disorder; OUD, opioid use disorder.

Use of MOUD during the 12-month period surrounding residential treatment stays followed a bell-shaped pattern (Figure 1). Specifically, rates were below 10% for all study years 6 months prior to residential treatment stays; increased gradually during the months preceding residential treatment stays; jumped up during the month of residential treatment stays; and then receded similarly strongly during subsequent months.

In regression analysis, the level of any MOUD treatment 6 months prior to the month of residential treatment stays increased by 2.7 percentage points between 2017 and 2021, by 28.5 percentage points during the month of residential treatment stays, and by 4.4 percentage points 5 months following the month of residential treatment stays (Table 1). The average number of months with any MOUD treatment during the 12-month period surrounding residential treatment stays was 1.04 in 2017; it increased by 0.81 months between 2017 and 2021.

**Table 1.** MOUD treatment across states, 2017-2021.

MOUD treatment	Level, 2017	Change 2017-2021
Rate, 6 months prior to stays	3.5	2.7***
Rate, month of stays	30.4	28.5***
Rate, 5 months following stays	4.0	4.4***
Number of months	1.04	0.81***

^****^ *P* < 0.001.

Source: Transformed Medicaid Statistical Information System Analytic Files 2016-2021.

Abbreviation: MOUD, medication for opioid use disorder.

Outcome levels were similar in waiver and non-waiver states in 2017 (Table 2). Changes in these outcomes were similar and did not differ significantly in waiver states and non-waiver states.

**Table 2.** MOUD treatment by waiver status, 2017-2021.

MOUD treatment	Waiver states		Non-waiver states		Relative change
	Level, 2017	Change, 2017-2021	Level, 2017	Change, 2017-2021	
Rate, 6 months prior to stays	3.6	1.9	1.5	10.4	−8.5
Rate, month of stays	30.4	28.2***	30.1	32.2***	−4.0
Rate, 5 months following stays	3.1	3.5***	2.8	13.6	−10.1
Number of months	1.06	0.69***	0.87	1.95*	−1.26

^*^ *P* < 0.05; ***P < 0.001.

Source: Transformed Medicaid Statistical Information System Analytic Files 2016-2021.

Abbreviation: MOUD, medication for opioid use disorder.

**Figure 2.**
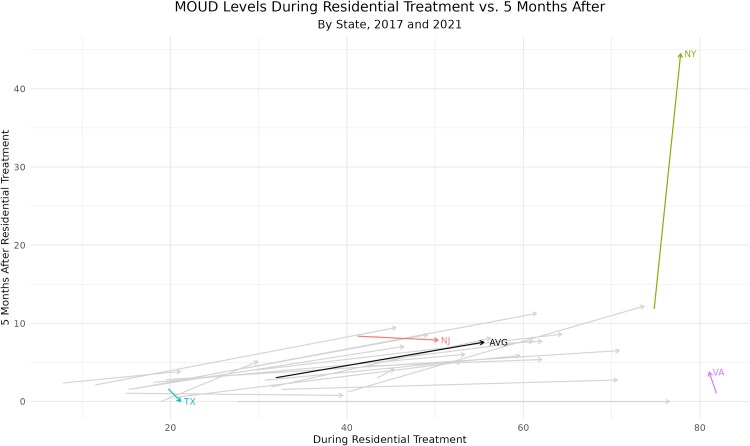
MOUD treatment during and 5 months following residential treatment stay, 2017-2021, by states. Notes: The figure shows levels of MOUD treatment during the residential stay (x-axis) and 5 months following the beginning of a residential treatment stay (y-axis) in 2017 and 2021. Arrows indicate change over time. Source: Transformed Medicaid Statistical Information System Analytic Files 2016-2021. Abbreviation: MOUD, medication for opioid use disorder.

Increases in MOUD treatment were similar across states, with a few exceptions (Figure 2). Most notably, MOUD treatment rates increased substantially in New York 6 months preceding residential treatment stays; more generally, MOUD treatment rates increased substantially in this state during the months preceding and following residential treatment stays (Appendix Section A-4 [To access the appendix, click on the Details tab of the article online.]). Three other states had somewhat unusual patterns: average MOUD treatment rates 5 months after residential treatment declined in New Jersey and Texas, and Virginia's MOUD rate during the month of residential treatment exceeded 80 percentage points in 2017 and declined slightly in subsequent years.

In sensitivity analyses, results were robust to excluding states with waivers approved in 2017 and 2021 or using a staggered regression specification (Appendix Section A-5 [To access the appendix, click on the Details tab of the article online.]). Excluding New York, a non-waiver state, from regressions assessing changes by waiver status yielded smaller estimates of increases in all outcomes among non-waiver states. However, relative changes between waiver and non-waiver states were still close to zero and not statistically significant. Finally, in regressions for each type of MOUD, increased use of buprenorphine fully accounted for the overall increase in MOUD treatment.

## Discussion

In this study, we examined changes in MOUD treatment during the 12-month period surrounding residential treatment stays. Across all study states, rates of MOUD during residential treatment increased substantially between 2017 and 2021, but the increase was not sustained during the months following stays. There was no positive association between SUD waiver adoption and increased use of MOUD treatment. Most states exhibited similar changes, with New York being the notable exception: it was the only state for which MOUD treatment increased substantially during the months surrounding residential treatment stays.

The substantial increase in MOUD treatment during residential treatment stays in both waiver and non-waiver states suggests that the waiver requirement has not been particularly effective. This result may be surprising given the positive association between waiver implementation and residential treatment facilities' willingness to provide MOUD for Medicaid patients.^12^ However, the number of Medicaid enrollees with OUD accessing residential treatment also increased with waiver implementation.^13^ Thus, the absolute number of Medicaid enrollees accessing MOUD in residential treatment settings may have increased more in waiver states compared to non-waiver states, despite a similar increase in the share of Medicaid enrollees receiving MOUD in residential treatment settings across these 2 groups of states. Moreover, some waiver states may have focused more on connecting Medicaid enrollees to MOUD in residential settings than others as part of their waiver, which could have resulted in variation across waiver states. Examining the combined role of waivers in increasing access to residential treatment and MOUD in this setting could be a fruitful venue for future research.

Approximately 2 out of 5 Medicaid members with OUD still did not receive MOUD treatment during their residential treatment stay in 2021. This finding may reflect common and ongoing barriers to MOUD treatment, notably stigma among patients, utilization management policies such as prior authorization requirements, and logistical challenges such as distance to opioid treatment programs (OTP).^20-22^ Continued efforts to reduce these barriers may further increase MOUD treatment in residential settings. States may also consider providing financial incentives to residential treatment providers for connecting patients to MOUD.

In supplemental analysis, we found that an increase in buprenorphine prescriptions entirely explains the increase in the overall use of MOUD in residential settings. Presumably, prescribing buprenorphine is logistically easier than arranging visits to an OTP to receive methadone treatment. Interviews with OTP providers could further help identify reasons why buprenorphine was the most common type of MOUD in this setting.

While more Medicaid members continued MOUD treatment after their residential treatment, the increase was muted, and most discontinued MOUD in the months afterwards. Care transitions from one setting to another can disrupt treatment adherence, and our results suggest that this may be an acute problem among Medicaid members accessing residential treatment stays. Further, we excluded Medicaid beneficiaries who disenrolled during the 12-month period surrounding residential treatment stays, who may have an even higher probability of discontinuation due to losing Medicaid coverage. Future efforts could investigate factors underlying this strong drop and how to best follow up on MOUD treatment after discharge from a residential treatment stay.

MOUD treatment rates were low 6 months prior to residential treatment stays in 2017 and only increased slightly over time. This finding contrasts with the strong increase in MOUD treatment among the overall Medicaid population with OUD. Apparently, those accessing residential care were much more disconnected from MOUD treatment compared to other Medicaid members with OUD, suggesting that they were a particularly vulnerable population that may be at risk of overdose and other adverse outcomes. Of note, rates of MOUD increased in the months preceding residential treatment. This may reflect patients' increased motivation to get treatment. A more in-depth examination of the treatment needs and barriers to accessing MOUD for this population could help more effectively connect them to MOUD independently of residential treatment stays.

New York stood out as the only state for which MOUD treatment rates increased substantially during the months surrounding residential treatment stays. By contrast, increases in MOUD treatment during the months surrounding residential treatment stays were fairly small in other states. Understanding how New York managed to connect this population to MOUD treatment could be valuable for other states interested in increasing MOUD treatment for people accessing residential services.

The average length of stay was approximately half a month; however, the length of stay varied. Future research may examine the relationship between length of stay and MOUD initiation and subsequent discontinuation. Such work may be able to show whether a longer length of stay could be beneficial in terms of connecting people to MOUD and establishing their treatment so that they have a higher chance of remaining on it their residential treatment stay.

## Supplementary Material

qxag175_Supplementary_Data
