# Supplementary material for: Opioid use disorder medication treatment in residential settings increased 2017-2021, but sustaining it following stays remains an issue
Source: Health Aff Sch. 2026 Aug 10;4(8):qxag175. doi: 10.1093/haschl/qxag175 (PMC13455005; doi:10.1093/haschl/qxag175)
Supplement: Supplementary file 1 [file qxag175_supplementary_data.zip › qxag175_Supplementary_Data.pdf]

The T-MSIS Analytic Files (TAF) Analysis Reporting Checklist

AcademyHealth

The TAF Analysis Reporting Checklist is designed to guide reporting of research using the TAF Medicaid claims data. It serves to promote transparency and reproducibility in TAF-based studies. For more information on the TAF Analysis Report Checklist and how it should be used, visit **TAFChecklist.org**

Category	Description	Location(s) in Manuscript Where Items Reported
**Data**		
Files, Years, Release Versions, and Data Extract	• Indicate which TAF files were used in the analysis (e.g., Demographic and Eligibility File, Inpatient File, Other Services File).<br>• Indicate which years of TAF data were included in the analysis.<br>• Indicate which file versions were included in the analysis (e.g., preliminary, release 1, release 2).<br>• Indicate whether the study drew from 100% TAF files or pre-specified extracts.	**Study Data and Methods / Data Source**
**Analytic Sample**		
Eligibility Criteria	• If applicable, describe what eligibility category codes were used to identify the study sample and whether they were used in combination with any other variables (e.g., age, receipt of specific medical services).	**Study Data and Methods / Study population**
Enrollment Span	• If applicable, indicate the minimum period of enrollment required for an enrollee to be included in the study sample and how the enrollment period was defined.	**Study Data and Methods / Study population**
Scope of Benefits	• If applicable, indicate whether the analysis included enrollees with full scope, comprehensive, or restricted benefits.	**Study Data and Methods / Study population**
Encounter Data	• Indicate whether the analysis excluded either fee-for-service or managed care enrollees; if managed care enrollees were excluded, define the criteria used to do so.<br>• Indicate which types of claims records (e.g., fee-for-service claims, service tracking claims, capitation payments; see variable CLM_TYPE_CD) were included in the analysis.	**Study Data and Methods / Study population**

The T-MSIS Analytic Files (TAF) Analysis Reporting Checklist

Category	Description	Location(s) in Manuscript Where Items Reported
**Data**		
Dual Eligibility	• Describe whether individuals dually enrolled in Medicare and Medicaid were included in or excluded from the study sample and, if applicable, how dual eligibility was defined.	**Study Data and Methods / Study population**
**State and Territory Exclusions**		
Criteria	• Indicate which states and/or territories were included (or excluded) from the analysis on the basis of data quality concerns.<br>• Indicate the criteria by which state exclusions were made, including measures, data sources, and thresholds.	**Study Data and Methods / Study population**
State Variation Table	• Consider including a statelevel table (which may appear in an appendix) summarizing the number of observations, means, medians, and missingness for key study measures.	
**Special Considerations**		
Spending	• Indicate which types of claims records (e.g., fee-for-service 3 claims, service tracking claims, capitation payments; see variable CLM_TYPE_CD) were included to measure spending.<br>• If including service-specific spending for managed care encounters, indicate how spending was imputed (payments from plans to health care facilities and professionals on encounter records are generally redacted).	**Not applicable**
Using TAF with Predecessor MAX Data	• Indicate if the analysis included data from the MAX files and, if so, for what years and which states.<br>• If applicable, include an exhibit examining trends in key measures by state over time and particularly during any transition from MAX to TAF.	**Not applicable**

Abbreviations: MAX (Medicaid Analytic eXtract), TAF (T-MSIS Analytic Files)

Considerations

The TAF Checklist includes reporting recommendations for TAF-based analyses. Authors of TAF-based research should indicate whether they followed the checklist in their manuscripts; an appendix table may be helpful to indicate whether and how the authors complied with each recommendation. Checklist items are recommendations, not prescriptions, and can be adapted to different study designs. Not all checklist items will apply to all studies. The TAF Checklist is not meant to be a substitute for broader reporting checklists (e.g., the **STROBE** reporting guideline for observational research or the **RECORD** reporting guideline for research using routinely collected health data). Rather, it is meant to complement those resources with guidance on TAF-specific methodological considerations.
